# Design and Test of a Low-Cost RGB Sensor for Online Measurement of Microalgae Concentration within a Photo-Bioreactor

**DOI:** 10.3390/s150304766

**Published:** 2015-02-26

**Authors:** Micaela Benavides, Johan Mailier, Anne-Lise Hantson, Gerardo Muñoz, Alejandro Vargas, Jan Van Impe, Alain Vande Wouwer

**Affiliations:** 1. BioSys, University of Mons, Boulevard Dolez 31, 7000 Mons, Belgium; E-Mails: micaela.benavides@umons.ac.be (M.B.); johan.mailier@fpms.ac.be (J.M.); anne-lise.hantson@umons.ac.be (A.-L.H.); 2. Unidad Académica Juriquilla, Instituto de Ingeniería, Universidad Nacional Autónoma de México, Blvd. Juriquilla 3001, Querétaro 76230, Mexico; E-Mails: GMunozM@ii.unam.mx (G.M.); AVargasC@ii.unam.mx (A.V.); 3. Department of Chemical Engineering, Catholic University of Leuven, 3001 Leuven, Belgium; E-Mail: jan.vanimpe@cit.kuleuven.be

**Keywords:** biomass concentration, microalgae, bioprocess, instrumentation, state estimation, monitoring, Luenberger observer, Arduino board

## Abstract

In this study, a low-cost RGB sensor is developed to measure online the microalgae concentration within a photo-bioreactor. Two commercially available devices, *i.e.*, a spectrophotometer for offline measurements and an immersed probe for online measurements, are used for calibration and comparison purposes. Furthermore, the potential of such a sensor for estimating other variables is illustrated with the design of an extended Luenberger observer.

## 1. Introduction

Research and applications of microalgae cultivation have experienced a remarkable increase in the last two decades due to a renewed interest in alternative energy sources, and the potential of microalgae to produce large quantities of neutral lipids (e.g., 20%−50% dry cell weight) for biodiesel production [1,2]. Besides, microalgae have a large spectrum of applications ranging from the production of pigments, cosmetics, animal fodder to wastewater treatment [2,3]. These ever increasing applications motivate the development of process monitoring and control, in order to improve process reliability and productivity. However, process instrumentation remains problematic, and it is usually not possible to measure online all the component concentrations of interest. An appealing alternative is therefore the development of a process model, and the design of an observer, or software sensor, blending the predictive capability of this dynamic model and the information from the available online sensors.

A classical model of microalgae cultivation under substrate limitation is the one originally proposed by Droop [4]. In [5], it has been demonstrated that observability is achieved when biomass is measured. In other words, other state variables, such as the extracellular concentration of substrate and the internal substrate quota, can be reconstructed online based on the process model and the available biomass measurement, provided that this measurement does not vanish (this is a requirement for having not only online information but also a properly operating photo-bioreactor). The biomass concentration therefore appears as a key measurement in order to set up a monitoring and control system.

Nowadays, conventional methods for measuring biomass include sample dry weight, direct count using microscopy [6], spectrophotometry [7], protein concentration [8], or fluorescence [9,10]. However, some of these methods can be time-consuming, destructive when they are based on sampling, and relatively costly in terms of acquisition costs or maintenance. Some recent developments include also a photo-electrochemical sensor [11] and the measurement of O_2_ generation [12].

In this context, there is a clear incentive for the development of low-cost sensors that would be appropriate for biomass online monitoring, and it is the purpose of this study to demonstrate the feasibility of a sensor based on an RGB color generator and a light sensor to detect the color of a surface.

RGB sensors have already been used in related applications, such as the measurement of wine or oil color by means of optical fiber [13–16], showing satisfactory results with a low cost implementation.

This paper is organized as follows. The next section introduces the material and methods used in this study. The data measured by the RGB sensor is correlated to the biomass concentration. In Section 3, the behavior of our proposed sensor is compared with that of a commercial optic online sensor, both working simultaneously in the same batch culture implemented in a photo-bioreactor for *Dunaliella tertiolecta*. The potential application of the RGB sensor in monitoring and control is also illustrated with the design of a software sensor (a Luenberger observer) for the extracellular and intracellular nutrient concentration. Finally some conclusions are drawn in Section 4.

## 2. Materials and Methods

In this work, we propose as a biomass online measurement method the use of a low-cost RGB light sensor to estimate the turbidity of the medium containing the microalgae. In this section, we describe the turbidity acquisition method, the luminance, turbidity and biomass relationships, and the materials used to implement the biomass sensor.

### 2.1. General Overview

**Figure 1.**
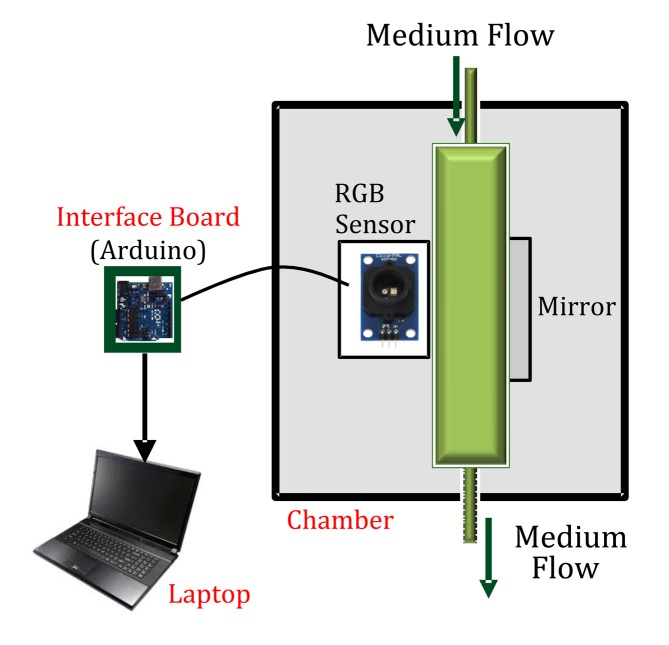
Sketch of the RGB sensor system.

The turbidity sensor consists of a sensing chamber, an interface board for data acquisition, and a personal computer for processing data. The sensing chamber is a dark (black) box that avoids the interference of external light from the environment with the sample, and comprises the main components: (a) an RGB light sensor; (b) a reflective material (mirror); and (c) a transparent plastic tube (containing a sample of the culture medium). The whole system is depicted in Figure 1.

The light sensor is composed of a color generator and a color sensor. Light is emitted by an LED, which delivers the three basic colors of the light: red, green and blue. The light is propagated through a plastic tube containing the culture medium with the microalgae, up to a mirror located on the other side of the plastic tube. The reflected light is then captured by the RGB color sensor, which sends the luminance of the medium as a combination of Red (R), Green (G) and Blue (B) components in digital format (from 0 to 1) to an external device.

### 2.2. Luminance, Turbidity and Biomass Relationships

The international standard recommendation ITU-R BT.709, used in video systems, defines the component weights R, G and B as a color space representation called YCbCr [17]. According to this standard, Y represents the luminance and it is calculated as a weighted sum of each color component shown in Equation (1), while Cb and Cr represent chroma components.

(1) Y=0.2126R+0.7152G+0.0722B

In the experimental setup under consideration, the luminance measurement *Y* is linked to the intensity of light *I* passing through the sample and reflected to the sensor. This intensity is of course linked to the turbidity of the sample, and in turn to the concentration of microalgae in the medium. The absorbance ABS_RGB_ of this medium can be estimated using the following Beer-Lambert law:

(2) $${\text{ABS}}_{\text{RGB}}=-\ln\left(\frac{I}{I_{0}}\right)$$

where *I* is the light intensity reflected through the medium and *I*_0_ is the light intensity reflected through a blank (deionized water).

The light absorbed by the culture medium can be experimentally correlated to the biomass concentration. In order to determine the correlation curve, the dry weight method can be used as a reference.

The biomass concentration can be linearly correlated to the absorbance, *i.e.*,

(3) $$\text{Biomass}\left[\text{mg}/\text{L}\right]={\text{ABS}}_{\text{RGB}}\cdot m+b$$

where *m* and *b* are the parameters obtained from the calibration process.

### 2.3. Implementation

The RGB light sensor selected in this implementation is the color sensor from Parallax, called the ColorPAL module, which incorporates an RGB LED and a TAOS TSL13T light-to-voltage converter [18] as the color sensor.

**Figure 2.**
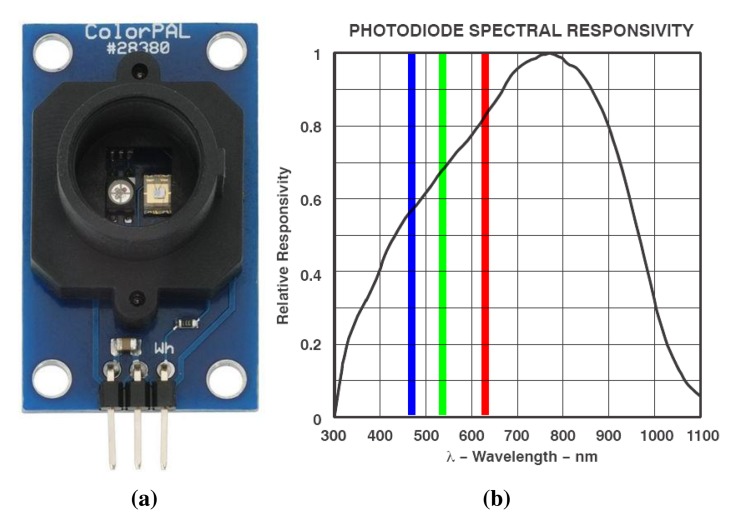
Picture of the ColorPAL module and its response. Picture of the ColorPal module (**a**) and its response (**b**).

The emitted light illuminates the sample contained in the tube and the color sensor TAOS TSL13T measures the amount of light reflected from the sample. This sensor is sensitive in the visible light spectrum, with a peak response at 780 nm, including the wavelengths of the RGB colors (red = 580 nm, green = 540 nm and blue = 450 nm). The use of the ColorPAL module as a color detection device is suitable mainly for solid surfaces, because the light is reflected back in proportion to the surface color. However, for liquid media, light reflection is not a good method to detect the color properties of the liquid. To alleviate this limitation, a mirror is placed on the opposite side of the sample, making the reflected light a better image of the medium turbidity. Figure 2 shows a picture of the ColorPAL module and the spectral response of the color sensor. The wavelengths of the RGB colors are also shown.

**Figure 3.**
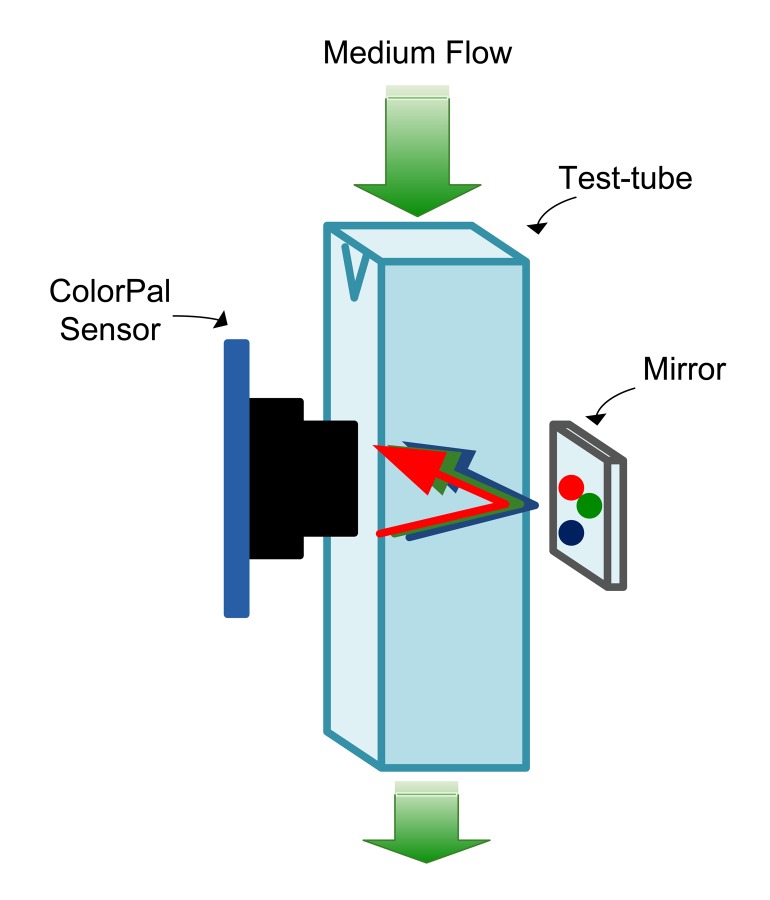
Sketch of the sensing space.

The sensing space is a transparent plastic tube adapted from a 4 mL test tube, originally designed for optical density measurements. It is made of polystyrene and has good optical properties for the visible wavelength range of 340–750 nm. A continuous flow of the culture medium occurs through the tube, which can be created using a peristaltic pump or by an air pump (using the airlift principle). The sensor is located on one side, and a mirror is placed at the opposite side to reflect the light from the LED, as shown in Figure 3. In order to avoid the interference of external light, a black and opaque plastic box encloses the sensing space.

The ColorPAL has a single data pin for bidirectional communications connected to an Arduino Uno board [19]. This board is based on an ATmega328 microcontroller, and it is used as the interface between the external computer and the color sensor. The ATmega328 microcontroller was programmed in the C-based Arduino language to receive the RGB parameters from the color sensor and to send them to the external PC using the RS-232 protocol. The data collected by the PC is used by a data acquisition software implemented in LabVIEW (National Instruments, 2010). The data acquisition software performs the following tasks:

(1) Read the RGB data measurement.
(2) Calculate the luminance using Equation (1).
(3) Calculate the absorbance using Equation (2).
(4) Calculate the biomass concentration using the correlation curve according to Equation (3).
(5) Record the data, wait for the next sample time and return to task 1.

This interface allows to record data online and to change in an interactive way the parameters such as the sampling interval.

This sensor can be built at a low cost, *i.e.*, about US$70, considering the cost of the Arduino board (30$), the ColorPAL Sensor (20$), the black box (5$) and other small components such as the test tube, tubing and mirror.

### 2.4. Calibration

The calibration procedure has to be repeated each time the test tube is replaced, and for each microalgal species under consideration. In this study, our reference method is the absorbance measurement obtained from the UV spectrophotometer Shimadzu UVmini 1240, which is related to biomass concentration using the dry weight measurement. The experimental protocol of the dry weight measurement consists of the following steps:

(1) A sample is centrifuged and filtered through Whatman GF/C glass fiber to separate the medium.
(2) The filter together with the algae biomass are dried in a furnace at 105 °C until a constant weight is achieved and are then cooled in a desiccator for 20 min.
(3) The weight of the sample is obtained using a precision scale.

This procedure is performed three times and the average value is considered.

The UV spectrophotometer allows spectral data acquisition in the wavelength range from 190 nm to 1100 nm [20]. As it was experimentally observed that the maximum absorbance of the *Dunaliella tertiolecta* culture medium was achieved at 680 nm, the absorbance measurements with the UV spectrophotometer shall be taken at that wavelength.

**Figure 4.**
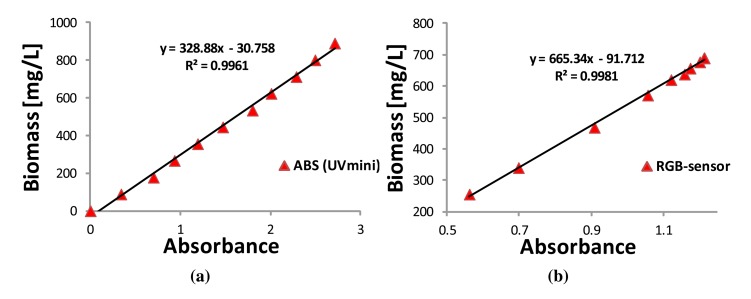
Calibration for the microalgal culture *Dunaliella tertiolecta*. (**a**) Relationship between biomass concentration and absorbance obtained from the spectrophotometer UVmini at 680 nm by 10 dilutions; (**b**) Relationship between biomass concentration and absorbance obtained online from the RGB sensor during the exponential growth.

A high concentration sample (891.11 mg/L according to the dry weight method) was taken from a microalgae culture and diluted in 10 steps, each time reserving aside part of the resultant solution and thus giving 11 samples. These diluted samples were analyzed and related with the corresponding dry weight measurements, and the results are presented in Figure 4a. As shown, a good linear correlation coefficient of R^2^ = 0.9961 is obtained for the full measurement range, and the equation defining the biomass is given by:

(4) $${\text{Biomass}}_{\text{UVmini}}\left[\text{mg}/\text{L}\right]=328.88\cdot {\text{ABS}}_{\text{UVmini}}-30.758$$

where ABS_UVmini_ is the absorbance measured by the UVmini sensor.

Note that this procedure is prone to errors due to the repeated dilutions, and that it probably gives an idealized view of the correlation since the samples contain the same cells (same age, same size distribution, same chemical composition) diluted in water. A better procedure would consist of independent samples with their variations inherent in the microalgae population.

To characterize the proposed RGB sensor, a culture of the microalgae *Dunaliella tertiolecta* was achieved, from which the absorbance was measured using the UVmini sensor and the RGB sensor. The culture started at 255 mg/L and grew until 690 mg/L of biomass concentration. During the exponential growth, 10 samples were taken at different times, in which their absorbances were measured by both sensors. In such samples, the reference biomass concentration was obtained by using the absorbance obtained from the UVmini sensor and Equation (4). Finally, a relation between the reference biomass concentration and the absorbance obtained from the proposed RGB sensor is established, and their results are depicted in Figure 4b. As shown, the linear correlation coefficient is R^2^ = 0.9981, and its equation is determined by:

(5) $${\text{Biomass}}_{\text{RGB}}\left[\text{mg}/\text{L}\right]=665.34\cdot {\text{ABS}}_{\text{RGB}}-91.712$$

where ABS_RGB_ is the absorbance measured by the proposed RGB sensor.

Table 1 shows some performance features of the proposed RGB sensor and the UVmini sensor. For the RGB sensor, the sensitivity is estimated from the slope of the curve determined by the relation of biomass concentration (abscissa) and absorbance (ordinate). The accuracy is calculated taking as a reference the biomass from the UVmini sensor. The precision is provided as a standard deviation of the biomass concentration estimated by the proposed sensor from 30 repeated measurements [21]. A similar procedure is followed to determine the sensitivity of the UVmini sensor; its accuracy is taken from the data sheet of manufacturer and expressed in mg/L, whereas the precision is evaluated from 10 repeated measurements.

## 3. Results and Discussion

**Figure 5.**
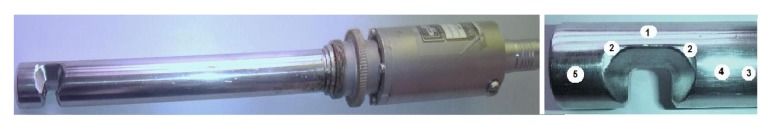
Optek sensor (http://www.optek.com/).

In this section, the performance of the proposed RGB sensor is evaluated against a commercial optical sensor, the Near Infrared (NIR) absorption probe (Optek, model ASD19-N). The main advantage of the Optek probe is that it can be used as an immersed probe in a photo-bioreactor (PBR), and it can take the measurements online. The measurement range of the Optek probe is from 0 to 4 concentration units (CUs). Here CU is a turbidity unit and, according to Optek, it is directly correlated to the absorbance. The image of this probe is shown in Figure 5. In the right subfigure, a schematic representation of the sensor is displayed where the different parts are indicated by numbers. An infrared LED (5) emits a light in the range of 840 nm to 910 nm. This light travels through a sapphire lens (2), the channel (1), and another sapphire lens (2), which in turn focuses the light as it passes through the channel. Then, the light passes through a daylight filter (4), which filters out any other sources of light. Finally, the light is captured by a detector (3), which converts it to a voltage.

**Figure 6.**
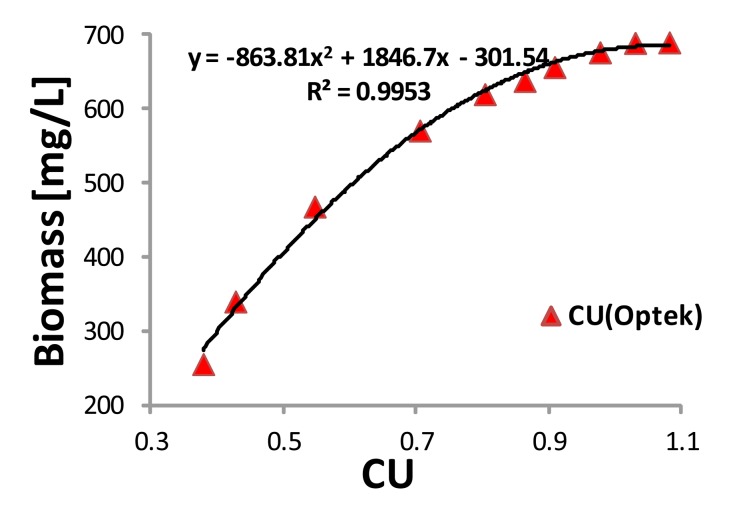
Correlation between the CU (Optek) and the dry biomass of the strain *Dunaliella tertiolecta*.

Following the same procedure as in the previous section, a calibration curve of the Optek sensor was obtained. Ten samples were also taken during the exponential growth and the biomass concentration indirectly obtained from the UVmini was used as a reference. Figure 6 shows the quadratic correlation between CU and biomass concentration with the correlation coefficient being R^2^ = 0.9953.

**Figure 7.**
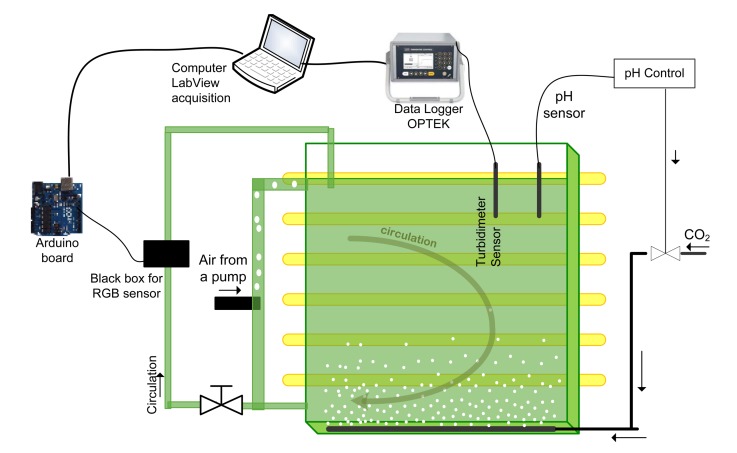
Schematic representation of the plant for microalgae culture.

To compare both sensors in normal PBR operation, batch experiments were performed for the culture of the microalgae *Dunaliella tertiolecta* using a Johnson medium [22]. As shown in Figure 7, these cultures were implemented in a flat sheet PBR of 13 L with a constant light source composed of a set of dimmable 18 W fluorescent tubes generally used in horticulture (Fluora 18 W/77, Osram) and with the main emitted wavelengths located in the visible spectrum (blue: 430 nm; red: 620 nm) to promote photosynthesis at a photon flux density of 150 *μ*mol · m^−2^ · s^−1^. The pH value was controlled by adding CO_2_ and was kept constant at 7. The cultures were performed in a laboratory environment where the temperature was kept between 23 °C and 28 °C. The injection of air at a rate of 0.1 L/min allowed medium mixing and circulation. The Optek probe was connected to a data logger that sent the CU information to the computer using a RS232 protocol, where they were handled using our in-house LabVIEW program. The information was collected every 5 min. On the other hand, the RGB sensor was placed on a recirculation pipe, where the liquid flow was ensured by the airlift principle.

**Figure 8.**
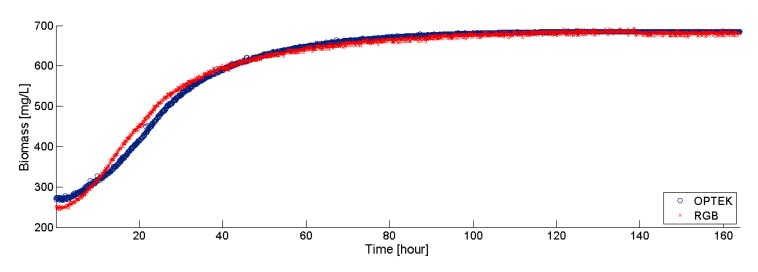
Data collection using an RGB sensor and Optek sensor in a microalgal culture.

The data collected during 163 h (at 5 min intervals) by both the RGB sensor and the Optek probe, are plotted in Figure 8. Note that both sensors show similar behaviors in the experiment. The biomass measurements show the typical exponential growth that characterizes the microalgal growth while substrate is still available. These results indicate that the proposed RGB sensor could be a viable alternative for the online monitoring of biomass in PBR. The main advantage of the RGB sensor is its extremely low cost, as compared with other commercial counterparts.

### 3.1. Potential RGB Sensor Applications in Monitoring and Control

A sensor of biomass concentration is of course of paramount importance for following the evolution of the culture online, but this sensor can also be used in combination with a process model to estimate other state variables, as long as observability conditions are satisfied.

In this section, we will consider as an application example, the Droop model [4], which is a classical model to describe substrate limitation in microalgal cultures.

The Droop model has three state variables: the concentration of the biomass *X*, the substrate *S*, and the intracellular quota *Q*. It is defined by the following dynamic mass balance equations:

(6) $$\begin{array}{ll} \dot{X} & =\mu \left(Q\right)X-DX\\ \dot{S} & =-\rho \left(S\right)X-DS+DS_{in}\\ \dot{Q} & =\rho \left(S\right)-\mu \left(Q\right)Q \end{array}$$

The dilution rate *D*(*t*) = *F_in_*(*t*)/*V*(*t*) is the ratio of the inlet flow rate to the volume of the culture. Here, we assume continuous operation with constant volume, *i.e.*, a chemostat. The absorption rate *ρ* (*S*) is defined by the Monod law:

(7) $$\rho \left(S\right)=\rho_{m}\frac{S}{S+K_{s}}$$

where *K_s_* is the half saturation constant for the substrate, and *ρ_m_* is the maximum inorganic nitrogen absorption rate. Droop proposed that the algal growth rate depends on the intracellular quota in the following way:

(8) $$\mu \left(Q\right)=\mu_{m}\left(1-\frac{Q_{0}}{Q}\right)$$

where *μ_m_* is the maximum growth rate, and *Q*_0_ is the minimal cell quota under which value the algae do not grow any more.

It has been shown in [5] that the Droop model is observable provided that the biomass evolution is measured and that it does not vanish. In other words, it is possible to estimate the substrate concentration and the intracellular quota online using a process model and the biomass measurement, so as to build a software sensor. In the following, the design of such a software sensor is exemplified.

Considering the Droop model as a nonlinear model (*ẋ* = *f*(*x*, *u*), *y* = *h*(*x*)), an extended Luenberger observer is defined as:

(9) $$\begin{array}{ll} \dot{x} & =f\left(\hat{x},u\right)+L\left(y-\hat{y}\right)\\ \hat{y} & =h\left(\hat{x}\right) \end{array}$$

where *x̂* is an estimate of the state vector and *L* is the observer gain. Defining the observation error as *e* = *x̂* − *x*, the dynamics of the error is:

(10) $$\dot{e}=f\left(\hat{x}+e,u\right)-f\left(\hat{x},u\right)-L\left(h\left(\hat{x}+e\right)-h\left(\hat{x}\right)\right)$$

Assuming that the difference of the functions *f* (*x̂* + *e*, *u*) − *f* (*x̂*, *u*) and *h* (*x̂* + *e*) − *h* (*x̂*) can be approximated to its partial derivative, the error dynamic is as follows:

(11) $$\dot{e}\approx \left(F\left(\hat{x},u\right)-L\left(\hat{x}\right)C\right)e$$

where *F* (*x̂*, *u*) = *∂* (*f* (*x̂*, *u*)) /*∂* (*x*), and *C* = *∂h*/*∂x*. As *X* is the unique state to be measured, the measurement matrix is *C* = [1 0 0]. Notice that since the matrix *F* (*x̂*, *u*) considers the trajectory of the states, such a matrix is a nonlinear function. The nonlinearities of this function can be modeled in terms of the time-varying parameter vector *θ* (*t*), so the matrix *F* (*θ*) is an affine matrix function of *θ* and the error dynamics can be redefined as *ė* ≈ *(F* (*θ*, *u*) − *LC*) *e*, where the vector *θ* (*t*) = [*θ*_1_⋯*θ*_5_] contains all nonlinear terms that are functions of the measurements [23].

The observer gain will be determined to guarantee the asymptotic stability of the system, using Lyapunov arguments and LMI-based (Linear Matrix Inequalities) techniques. The quadratic Lyapunov function is:

(12) $$V\left(t\right)=V\left(e\right)=e^{T}Pe$$

where *P* = *P^T^* > 0, *V* (*e*) > 0, *V̇* (*e*) < 0 with time derivative *V̇* (*e*) = *ė^T^Pe* + *e^T^Pė*. Including the error dynamics in this latter expression, it can be written as:

(13) $$\begin{array}{cc} \dot{V}\left(e\right) & =e^{T}{\left(F\left(\theta \right)-LC\right)}^{T}Pe+e^{T}P\left(F\left(\theta \right)-LC\right)e\\ \dot{V}\left(e\right) & =e^{T}\left(F{\left(\theta \right)}^{T}P-C^{T}L^{T}P+PF\left(\theta \right)-\text{PLC}\right)e \end{array}$$

Imposing *V̇* (*e*) < 0 and *R* = *L^T^P*, the following set of inequalities guarantee system stability :

(14) $$\{\begin{array}{ll} P & >0\\ F{\left(\theta \right)}^{T}P-C^{T}R+PF\left(\theta \right)-R^{T}C & <0 \end{array}$$

The observer gain *L* = *P*^−1^*R^T^* is obtained by solving this set of LMIs for a known polytope where the vector *θ* is assumed to be defined. The extended Luenberger observer can be defined as:

(15) $$\begin{array}{ll} \dot{\hat{X}} & =\mu \left(\hat{Q}\right)\hat{X}-D\hat{X}+L_{1}\left(X-\hat{X}\right)\\ \dot{\hat{S}} & =-\rho \left(\hat{S}\right)\hat{X}-D\hat{S}+D\cdot S_{in}+L_{2}\left(X-\hat{X}\right)\\ \dot{\hat{Q}} & =\rho \left(\hat{S}\right)-\mu \left(\hat{Q}\right)\hat{Q}+L_{3}\left(X-\hat{X}\right) \end{array}$$

The partial derivative matrix can be computed as follows:

(16) $$F\left(\hat{x},u\right)=\left[\begin{array}{ccc} \mu_{m}\left(1-\frac{Q_{0}}{\hat{Q}}\right)-D & 0 & \frac{\mu_{m}\hat{X}Q_{0}}{{\hat{Q}}^{2}}\\ -\rho_{m}\frac{\hat{S}}{\hat{S}+K_{s}} & -\rho_{m}\frac{K_{s}\hat{X}}{{\left(\hat{S}+K_{s}\right)}^{2}}-D & 0\\ 0 & \rho_{m}\frac{K_{s}}{{\left(\hat{S}+K_{s}\right)}^{2}} & -\mu_{m} \end{array}\right]$$

In order to obtain the *F* (*x̂*, *u*) matrix as a function of *θ* and *u*, the nonlinearities are grouped in *θ* = [ *θ*_1_ *θ*_2_ *θ*_3_ *θ*_4_ *θ*_5_ ], where:

$$\begin{array}{lll} \theta_{1}=\frac{\rho_{m}K_{s}X}{{\left(S+K_{s}\right)}^{2}}; & \theta_{2}=\frac{\rho_{m}K_{s}}{{\left(S+K_{s}\right)}^{2}} & \\ \theta_{3}=\frac{\rho_{m}S}{S+K_{s}}; & \theta_{4}=\frac{\mu_{m}XQ_{0}}{Q^{2}}; & \theta_{5}=\mu_{m}\left(1-\frac{Q_{0}}{Q}\right) \end{array}$$

The initial conditions and the parameters considered in this work are shown in Table 2. The parameter values were adapted from [24].

For this implementation, we define the polytope of admissible values of the state variables based on a preliminary system analysis: *χ* = {50 ≤ *X* ≤ 1000; 0.65 ≤ *S* ≤ 50; 0.06 ≤ *Q* ≤ 0.12}. Considering the range values of *χ*, the 2^5^ vertices that define the polytope are calculated. Finally, the LMIs are solved using the parser YALMIP [25] and the solver SeDuMi [26] in MATLAB, obtaining the following result for the observer correction gain *L* = *P*^−1^ *R^T^*.

$$L=\left[\begin{array}{l} 1.53e6\\ 1.83\\ -4.65 \end{array}\right]$$

**Figure 9.**
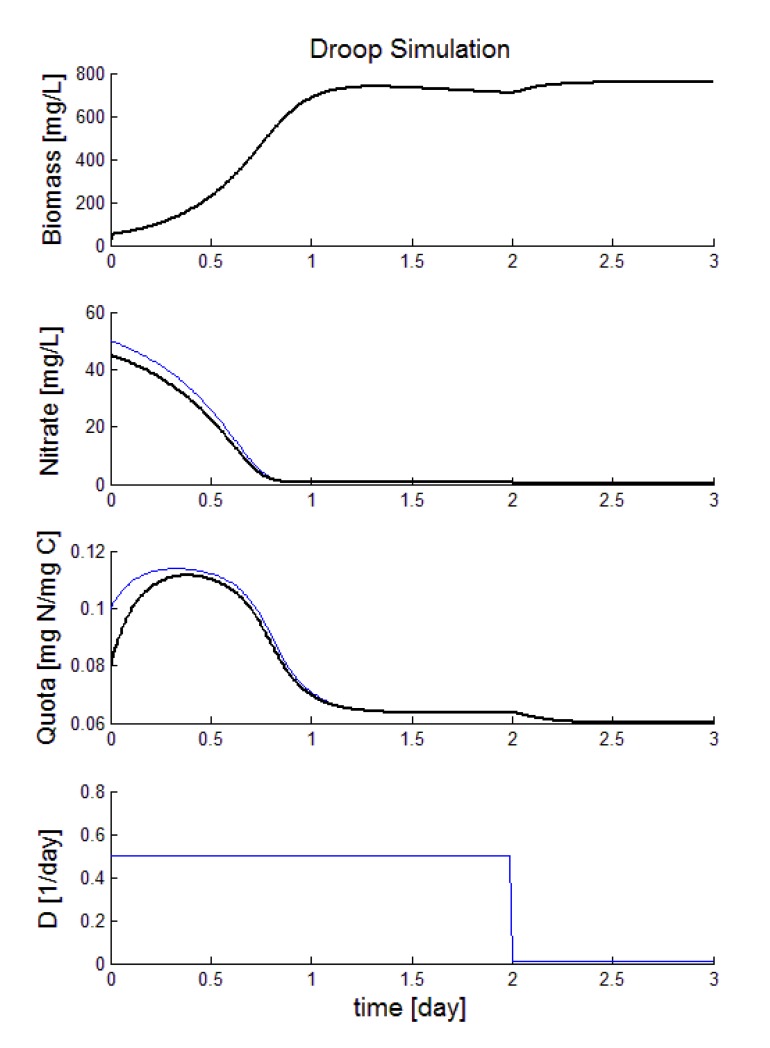
Estimated states in a microalgal culture.

The estimated states are plotted in Figure 9. The black line represents the real system and the blue one represents the estimated states. In our simulation the dilution rate is 0.5 day^−1^ during the firsts two days. However, in the second day the observer is also tested when the dilutions rate goes to zero. Notice that the observer achieves satisfactory values for the nitrate and quota concentrations just using the measurements of the biomass concentration.

## 4. Conclusions

In this paper, a low-cost online biomass concentration sensor based on a commercial RGB light sensor is proposed. In order to characterize our proposed sensor, the biomass concentration of the microalgae culture obtained by a commercial UV spectrophotometer is used as a reference. Then, the calibration procedure is presented and the sensitivity, accuracy and precision are evaluated. We compare our proposal with a commercial online sensor working in parallel in a microalgae culture implemented in a PBR. The results show that our proposal may be a suitable solution for online biomass concentration measurement. Finally, an example of its potential application in monitoring and control process is detailed. Future works will consider the use of the RGB sensor in a lab-scale process for microalgae cultivation.

## Figures and Tables

**Table 1.** Features of the proposed RGB sensor and the UVmini sensor.

	**RGB**	**UVmini**
Sensitivity (slope)	0.0015 Abs/mgL^−1^	0.003 Abs/mgL^−1^
Accuracy (Absolute error)	6.91 mg/L	1.64 mg/L
Precision (standard deviation)	2.26 mg/L	1.23 mg/L

**Table 2.** Initial conditions and parameters.

**Initial Conditions**	**Parameters**
*X*_(0)_ = 50 mgC L^−1^	*ρ_m_* = 0.526 mgN mgC^−1^d^−1^
*S*_(0)_ = 50 mgN L^−1^	*K_s_* = 10 mgN L^−1^
*Q*_(0)_ = 0.1gN gC^−1^	*μ_m_* = 7.71 d^−1^
	*Q*_0_ = 0.06 mgN mgC^−1^
	*S_in_* = 40 mg L^−1^ and *D* = 0.5 d^−1^
